# Dispersion-free extraction of In(III) from HCl solutions using a supported liquid membrane containing the HA324H^+^Cl^−^ ionic liquid as the carrier

**DOI:** 10.1038/s41598-020-70968-1

**Published:** 2020-08-17

**Authors:** Francisco José Alguacil, Félix Antonio López

**Affiliations:** National Center for Metallurgical Research (CENIM), Spanish National Research Council (CSIC), Avda. Gregorio del Amo, 8, 28040 Madrid, Spain

**Keywords:** Chemical engineering, Green chemistry

## Abstract

By reaction of HCl and the tertiary amine HA324, an ionic liquid denoted HA324H^+^Cl^−^ was generated and used in the transport of indium(III) from HCl solutions. Metal transport experiments were carried out with a supported liquid membrane, and several variables affecting the permeation of indium(III) across the membrane were tested: stirring speed, metal and acid concentrations in the feed solutions and the carrier concentration in the supported organic solution. The metal transport results were also compared with those obtained using different carriers in the solid support. A model that described indium(III) transport across the membrane was proposed, and the corresponding diffusional parameters were estimated.

## Introduction

Indium is classified by the European Commission as a critical raw material both for its economics and high supply risk, i.e., an expected increase in its demand over the next years. The above is a consequence of its special properties and wide range of industrial applications, i.e., liquid–crystal displays (LCDs), electronics, catalysts, etc. Thus, the recovery of this element from the above sources is an important target, and several technologies have been proposed to resolve this issue^1–5^, including ion-exchange^6^, adsorption^7^, cementation^8^, liquid–liquid extraction^9–11^, liquid membranes^12^ and a process that uses a sequence of steps: leaching-distillation-refluxing in SOCl_2_^13^. Moreover, the use of bioleaching with *A. thiooxidans* and *A. ferrooxidans* has been proposed in the treatment of LCD panels^14,15^; however, it is assumed that bioleaching processing has a higher environmental impact than some chemical processes due to its long duration and high electricity consumption^16^. In addition, indium is considered a hazardous element due to its carcinogenic character^17^. Its removal from residual aqueous solutions, i.e., that resulting from a number of the above processes, is of the foremost importance, and liquid membranes must be considered a technology suitable for the recovery of metals and other solutes present in wastewaters. Liquid membranes have presented a series of operational advantages over other separation technologies, e.g., electrophoresis. (i) The use of solvent extraction in the treatment of solutions containing metal at a few mg/L is not recommended; these concentrations are perfectly compatible with the use of liquid membranes. (ii) In liquid membranes, processing the extraction/stripping steps occurs simultaneously, whereas in solvent extraction, ion exchange with resins and adsorption processes, the above sequence occurs in two separate steps. Supported liquid membranes in various configurations, which are included in these liquid membrane operations, are useful for this task. Before the technology is scaled up in the form of hollow fibre modules, the investigation of a given system using a supported liquid membrane in a flat-sheet configuration is necessary to obtain information about the mass transfer processes involved in membrane operation. The key of the operation is the carrier used to transport the metals, and ionic liquids are such carriers. Ionic liquids are a group of chemicals whose properties identify them as *green solvents*, which among other uses^18–20^, makes them suitable for the removal of metals from aqueous solutions^21–26^.

As a part of the investigations carried out by our group related to the removal of indium(III) using liquid membrane technologies^12^, this work presented results for the removal of indium(III) from HCl solutions using a flat-sheet supported liquid membrane (FSSLM) containing the ionic liquid HA324H^+^Cl^−^ as the carrier. Different hydrodynamic and chemical variables affecting the indium(III) transport process were investigated.

## Results

### Influence of the stirring speed in the source phase on indium(III) permeation

In every separation technology, i.e., liquid membranes, ion exchange or adsorption, and when working in batch conditions, it is necessary to experimentally determine the best hydrodynamic conditions to ensure maximum solute transport, adsorption, etc. Thus, in the present system, the influence of the stirring speed in the source phase on metal permeation was investigated by the use of source phases containing 0.01 g/L In(III) in 2 M HCl and a receiving solution of 0.1 M HCl. The membrane phase was 0.23 M ionic liquid in Solvesso 100 solution supported in a Durapore GVHP4700 solid support. Table 1 shows the results from these experiments.

**Table 1:** Influence of stirring speed on the permeability (P) of indium(III).

Stirring speed (rpm)	P × 10^3^ (cm/s)
375	1.5
500	1.9
750	2.9
1000	1.5
1500	1.0

*P* value, as in the rest of the work, calculated as in Eq. (12).

### Influence of the stirring speed in the receiving phase on In(III) permeation

In the present work, the receiving phase was 0.1 M HCl because previous data^11^ indicated that this solution was a good strippant for this system. Experiments carried out with this solution, as the receiving phase, with the same source and membrane phases as above and a 750 min^-1^ stirring speed for the source phase, indicated that in the 500–750 min^-1^ range of stirring speeds applied to the receiving phase, there was no appreciable difference in the transport of the metal. Thus, stirring speeds of 750 and 500 min^-1^ in the source and receiving phases were selected for further experiments.

### Influence of HCl concentration in the source phase on indium(III) transport

**Figure 1:**
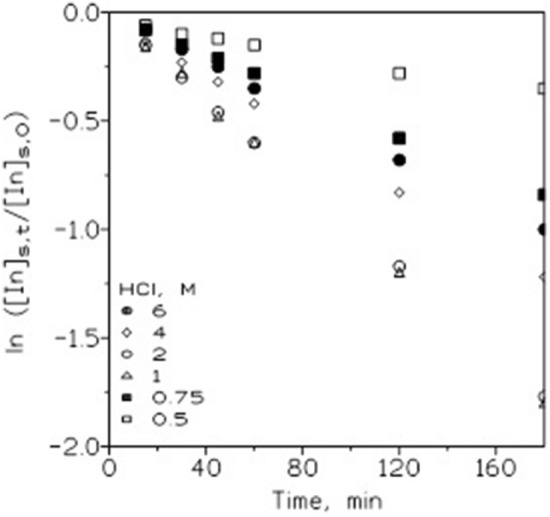
Influence of HCl concentration in the source phase on In(III) transport. The stirring speeds of the source and receiving phases were 750 and 500 min-1, respectively.

The variation of the HCl concentration in the source phase on In(III) permeation was investigated using the same receiving and membrane phases as above, with the source phase of 0.01 g/L In(III) in different HCl media. The results of the experiments are shown in Fig. 1 by plotting ln ([In]_s,t_/[In]_s,0_) versus time, where [In]_s,t_ and [In]_s,0_ are the metal concentrations in the source phase at some time and the initial time, respectively.

### Influence of the carrier concentration on In(III) permeation

**Figure 2:**
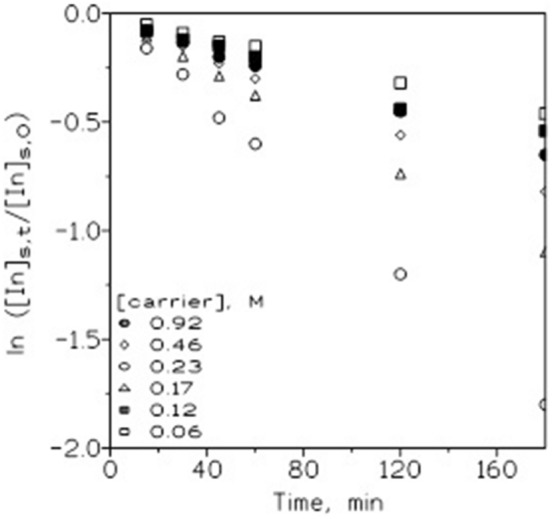
Influence of the carrier concentration on In(III) transport. Source phase: 0.01 g/L In(III) in 1 M HCl. Membrane phase: different concentrations of the ionic liquid in Solvesso 100 in GVHP4700 supports. Receiving phase: 0.1 M HCl.

It was mentioned above that the carrier solution is the key for liquid membrane operation since a support with no carrier phase in it does not transport metal. Figure 2 shows the results when 0.01 g/L In(III) in a 1 M HCl source phase was transported across a membrane containing organic phases with different concentrations of the ionic liquid in Solvesso 100 and a 0.1 M HCl solution was used as the receiving phase.

### Influence of the initial indium(III) concentration in the source phase on the metal permeation and flux

These results were obtained using membrane phases containing 0.23 M ionic liquid in Solvesso 100 that was supported in a GVHP4700 support and a receiving phase of 0.l M HCl. The source phases had various In(III) concentrations (0.01–0.1 g/L) in 1 M HCl. Table 2 presents these results.

**Table 2:** Variation in the indium(III) permeation coefficient (P) and flux (J) with variation in the initial metal concentration in the source phase.

Initial In(III) concentration (g/L)	P × 10^3^ (cm/s)	J × 10^10^ (mol/cm^2^ s)
0.01	3.0	2.6
0.05	0.82	3.6
0.1	0.72	6.3

The stirring speeds in the source and receiving phases were 750 and 500 min^-1^, respectively.

### Influence of the support characteristics on indium(III) transport

The results derived with the GVHP4700 support were compared with those obtained when the HVHP4700 support was used as a solid support in this system, with all the experimental variables the same as in Fig. 2, except that in this case, the membrane phase was a 0.23 M carrier in Solvesso 100 solution that was supported in the HPVP and GVHP solid supports.

### Indium(III) transport using different carriers

The transport of indium(III) was compared when different carriers were used. In this case, the source phase was 0.01 g/L In(III) in 1 M HCl, and the receiving phase was 0.1 M HCl. The membrane phases were 0.17 M carrier in Solvesso 100 solutions supported in the GVHP4700 solid support, and the stirring speeds applied to the source and receiving phases were 750 and 500 min^-1^, respectively.

These transport results, together with the indium(III) recovery in the receiving phase, are summarized in Table 3.

**Table 3:** Results of In(III) transport and metal recoveries in the receiving phase using different carriers.

Carrier	P (cm/s)	^b^In recovery (%)
Cyphos IL102	1.2 × 10^–3^	99
Cyphos IL101	1.4 × 10^–3^	99
Cyanex 923	8.5 × 10^–4^	99
Tributylphosphate	4.1 × 10^–4^	6
^a^Primene JMT	3.1 × 10^–4^	Nil
Aliquat 336	2.9 × 10^–4^	50
^a^Hostarex A324	1.8 × 10^–3^	83
2-Ethyl-1-hexanol	4.2 × 10^–7^	Nil
Isopentyl-methylketone	3.9 × 10^–4^	Nil

^a^Acting as the corresponding quaternary ammonium salt.

^b^After 3 h, and with respect to the metal transported from the source to the membrane phases.

## Discussion

It was shown^11^ that the extraction of indium(III) from hydrochloric acid media by the ionic liquid HA324H^+^Cl^−^ dissolved in Solvesso 100 (ExxonMobil ) occurs via the equilibrium

1 $$InCl_{{4_{aq} }}^{ - } + H324H^{ + } Cl_{org}^{ - } \Leftrightarrow HA324H^{ + } InCl_{{4_{org} }}^{ - } + Cl_{aq}^{ - }$$

where the subscripts org and aq refer to the organic and aqueous phases, respectively. Thus, extraction occurred when the equilibrium was shifted to the right, and metal stripping shifted the equilibrium to the left. It was also determined that the value of the equilibrium constant value for the above equilibrium was 10.96 in 1 M HCl medium.

Some authors^27^ considered that the extraction of indium(III) occurred via the extraction of InCl_3_ species and the formation of R + InCl_4_^−^ species in the organic phase, where R + represents the cationic moiety of the ionic liquid (in the chloride form), that is:

2 $$InCl_{{3_{aq} }} + R^{ + } Cl_{org}^{ - } \Leftrightarrow R^{ + } InCl_{{4_{org} }}^{ - }$$

However, these same authors also argued that the extraction of In(III) can be attributed to both reactions: ion exchange (as in Eq. (1)) and extraction of InCl_3_, as mentioned above.

Accordingly with the data presented in Table 1, the indium(III) permeation increased from 375 to 750 min^-1^ and then decreased, which is attributable to a continuous decrease in the source phase boundary layer thickness with increasing stirring speed in this phase. A limiting permeability value was obtained at 750 min^-1^. At this point, the system reached a minimum in the thickness of the aqueous source layer, and indium(III) transport was maximized; thus,

3 $$P_{\lim } = \frac{{D_{aq} }}{{d_{aq} }}$$

where D_aq_ is the metal diffusion coefficient in the aqueous source phase (averaging a value of 10^–5^ cm/s), d_aq_ is the thickness of the aqueous source layer, and P_lim_ is 2.9 × 10^–3^ cm/s. The value of d_aq_ is estimated to be 3.4 × 10^–3^ cm. Thus, this value represented the minimum thickness of the stagnant source phase layer, considering the experimental conditions used in this work. It is also shown in Table 1 that at stirring speeds above 750 min^−1^, the metal permeation decreased, which was caused by the turbulence caused by the stirring speed and the likely progressive instability of the liquid membrane.

Figure 1 shows that the indium permeation increased with increasing HCl concentration in the source solution up to 1–2 M and then decreased at higher HCl concentrations in this phase, which was probably attributable to the increase in the aqueous ionic strength and/or increases in the ion population in this phase (population growth effect), which often decreases metal permeation^28^.

Moreover, the percentage of indium(III) recovered in the receiving phase was, after three hours, approximately 90% when 1 or 2 M HCl solution was used.

From the results presented in Fig. 2, it was shown that the maximum metal permeability (3.0 × 10^–3^ cm/s) was reached when a carrier concentration of 0.23 M was used in the membrane phase. The above was attributable to the fact that, at low carrier concentrations, diffusion of the indium(III)-carrier complex across the liquid membrane governed the rate, but at the maximum indium(III) permeation, diffusion of indium(III) across the source phase boundary layer is the rate-determining process. According to Eq. (3), d_aq_ was estimated as 3.3 × 10^–3^ cm, a value that matched perfectly with that previously obtained with 2 M HCl concentration in the source phase.

When higher carrier concentrations, i.e., 0.46 and 0.92 M, were used in the membrane phase, increasing the organic phase viscosity decreased metal transport, which was attributable to an increase in the membrane resistance.

From results showed in Table 2, it was observed that, first, the permeation coefficient, P, decreased as the initial metal concentration in the source phase increased, and second, the initial metal flux increased with increasing initial indium(III) concentration in this phase.

The decrease in metal permeation with the increasing indium(III) concentration may occur because the membrane pores become saturated with increasing metal concentrations, whereas the flux results were logical since the flux varies with metal concentration:

4 $$J = P\left[ {In} \right]_{s,0}$$

Thus, the flux value increased with increasing metal concentration in the source phase. According to the flux values shown in Table 2, in the range of indium(III) concentrations used in this work, the permeation process seemed to be controlled by the diffusion of the metal species.

The percentage of metal recovered in the receiving phase varied between 90 and 99% (after 3 h) with respect to the metal transported from the source to the membrane phase for the three initial indium(III) concentrations used in this work.

When using GVHP or HVHP supports, the initial fluxes (Eq. 4) obtained from these experiments were 2.6 × 10^–10^ mol/cm^2^ s and 1.7 × 10^–10^ mol/cm^2^ s for both supports, respectively; thus, all the characteristics but the pore size (2.2 × 10^–3^ cm for GVHP versus 4.5 × 10^–3^ cm for HVHP) were similar for both supports. This last characteristic dominated the metal transport, and apparently, the smaller the pore size was, the greater the flux value with respect to the indium(III) recovery in the receiving phase (91% for GVHP and 59% for HVHP supports after 3 h and, as above, with respect to the metal transported from the source to the membrane phases).

From the results presented in Table 3 and under the present experimental conditions, the best transport results were obtained with the ionic liquid HA324H^+^Cl^-^, followed by the two ionic liquids Cyphos IL101 and Cyphos IL102, derived from a phosphonium salt. The worst transport result was obtained when 2-ethyl-hexanol was the carrier for indium transport. For the carriers derived from quaternary ammonium, the order found was tertiary amine (Hostarex A324) > Aliquat 336 > primary amine (Primene JMT); in the case of phosphorous derivatives, phosphonium salts > phosphine oxides > phosphoric ester; and for organics containing C–O bonds, ketone > alcohol. Apparently, indium(III) transport is closely related to liquid–liquid extraction using these same extractants. In fact, the liquid–liquid extraction of metal-chloride complexes is favoured when anion exchange equilibria are responsible for metal extraction, that is, as in the case of phosphonium salts and the tertiary amine. Neutral phosphorus derivatives, such as Cyanex 923 and TBP, extracted metals via the solvation of neutral metal species by donation of an electron pair from the oxygen of the P=O bond to the metal species, and in these particular cases, phosphine oxides had greater electron-donor properties than the phosphoric ester TBP. Alcohols and ketones also extracted metals via solvation of neutral metal species, and in these cases, it occurred via the donation of an electron pair (the oxygen atom in the C=O bond) in the case of the ketones or by bonding via the OH group of the alcohols and water molecules present in the metal complex.

The ranking of indium recovery in the receiving phase was not as clear; however, near quantitative In(III) recovery was achieved with Cyphos IL101, Cyphos IL102 and Cyanex 923, while worse recovery occurred with the HA324H^+^Cl^-^ ionic liquid. However, greater percentages of In(III) recovery can be obtained in this phase (see "Influence of the initial indium(III) concentration in the source phase on the metal permeation and flux" and "Influence of the support characteristics on indium(III) transport" sections). The 0.1 M HCl solutions were not good receiving media for the primary amine (acting as the corresponding ammonium form), the ketone and the alcohol.

Under the present experimental conditions, the best transport results were obtained with the ionic liquid HA324H^+^Cl^-^, followed by the two ionic liquids Cyphos IL101 and Cyphos IL102, derived from a phosphonium salt. The worst transport result was obtained when 2-ethyl-hexanol was the carrier for indium transport. For the carriers derived from quaternary ammonium, the order found was tertiary amine (Hostarex A324) > Aliquat 336 > primary amine (Primene JMT); in the case of phosphorous derivatives, phosphonium salts > phosphine oxides > phosphoric ester; and for organics containing C–O bonds, ketone > alcohol. Apparently, indium(III) transport is closely related to liquid–liquid extraction using these same extractants. In fact, the liquid–liquid extraction of metal-chloride complexes is favoured when anion exchange equilibria are responsible for metal extraction, that is, as in the case of phosphonium salts and the tertiary amine. Neutral phosphorus derivatives, such as Cyanex 923 and TBP, extracted metals via the solvation of neutral metal species by donation of an electron pair from the oxygen of the P=O bond to the metal species, and in these particular cases, phosphine oxides had greater electron-donor properties than the phosphoric ester TBP. Alcohols and ketones also extracted metals via solvation of neutral metal species, and in these cases, it occurred via the donation of an electron pair (the oxygen atom in the C=O bond) in the case of the ketones or by bonding via the OH group of the alcohols and water molecules present in the metal complex.

The ranking of indium recovery in the receiving phase was not as clear; however, near quantitative In(III) recovery was achieved with Cyphos IL101, Cyphos IL102 and Cyanex 923, while worse recovery occurred with the HA324H^+^Cl^-^ ionic liquid. However, greater percentages of In(III) recovery can be obtained in this phase (see see "Influence of the initial indium(III) concentration in the source phase on the metal permeation and flux" and "Influence of the support characteristics on indium(III) transport" sections). The 0.1 M HCl solutions were not good receiving media for the primary amine (acting as the corresponding ammonium form), the ketone and the alcohol.

The experimental results derived from this work, allowed to the estimation of the diffusional parameters involved in the present system. According to Eq. (1), the equilibrium constant of the reaction can be written as:

5 $$K = \frac{{\left[ {HA324H^{ + } InCl_{4}^{ - } } \right]_{org} \left[ {Cl^{ - } } \right]_{aq} }}{{\left[ {InCl_{4}^{ - } } \right]_{aq} \left[ {HA324H^{ + } Cl^{ - } } \right]_{org} }}$$

Following the same reasoning published elsewhere^29^, an expression for the permeability coefficient can be written as:

6 $$P = \frac{{K\left[ {HA324H^{ + } Cl^{ - } } \right]_{org} \left[ {Cl^{ - } } \right]_{aq}^{ - 1} }}{{\Delta_{org} + \Delta_{aq} \left( {K\left[ {Cl^{ - } } \right]_{aq}^{ - 1} \left[ {HA324H^{ + } Cl^{ - } } \right]_{org} } \right)}}$$

where Δ_aq_ and Δ_org_ are the transport resistance related to diffusion in the source and membrane phases, respectively. This expression combines the diffusional and equilibrium parameters involved in the transport of indium(III) from HCl solutions across a membrane supporting the ionic liquid. Then,

7 $$\frac{1}{P} = \Delta_{aq} + \Delta_{org} \frac{1}{{K\left[ {Cl^{ - } } \right]_{aq}^{ - 1} \left[ {HA324H^{ + } Cl^{ - } } \right]_{org} }} = \Delta_{aq} + \Delta_{org} \frac{1}{b}$$

Thus, a plot of 1/P versus 1/b for various carrier concentrations in the membrane phase and 1 M Cl^−^ concentration in the source solution may result in a straight line with an intercept and a slope that can be used to estimate the transport resistance in the source phase (Δaq = 166 s/cm) and in the membrane phase (Δorg = 0.84 s/cm), respectively. The estimated value of the membrane diffusion coefficient is:

8 $$D_{org} = \frac{{d_{org} }}{{\Delta_{org} }}$$

The membrane diffusion coefficient was 1.5 × 10^–2^ cm^2^/s here. In the above expression, d_org_ is the thickness of the membrane phase (see Sect. 3).

The diffusion coefficient of the indium(III)-ionic liquid complex in the bulk organic phase can be estimated by^30^:

9 $$D_{b,org} = D_{org} \frac{{\tau^{2} }}{\varepsilon }$$

which gave the D_b,org_ value 5.6 × 10^–2^ cm^2^/s. D_org_ had a lower value than D_b,org_, which may be caused by the diffusional resistance due to the thickness of the membrane between the source and receiving phases.

Moreover, an apparent diffusion coefficient for indium(III) can be estimated as:

10 $$D_{org}^{a} = \frac{{Jd_{org} }}{{\left[ {carrier} \right]_{org} }}$$

When the constant carrier concentration was assumed to be 0.23 M, the value of this coefficient was 1.3 × 10^–8^ cm^2^/s.

## Methods

The tertiary amine Hostarex A324 (Hoechst) has the active group tri-isooctyl amine and was used as the organic precursor of the ionic liquid. The inorganic moiety was hydrochloric acid, and the ionic liquid was generated by reaction of both^11^. Other carriers used in this investigation had the composition shown in Table 4. All were used without further purification. Other chemicals used in the work were G.R. quality, except for the organic diluent Solvesso 100 (99% aromatics), which was obtained from Exxon Chem., Iberia, and was also used without further purification. Though many authors claimed that they used ionic liquids without dilution, the truth was that in most, if not all, cases, an organic diluent was needed, and its use facilitated the liquid–liquid extraction operation because, among other reasons:

(i) It decreased the high viscosity of the ionic liquid and, thus, facilitated phase disengagement in the settler, and.
(ii) The range of carrier concentrations was adequate for any particular use. The use of an excess of carrier was avoided in the process, which gave the benefits of decreasing its financial cost and favouring metal transport, e.g., in this investigation, using an excess of carrier decreased indium(III) permeability.

**Table 4:** Chemical compositions and sources of the carriers.

Carrier	Chemical composition	Source
Cyphos IL102	Trihexyl tetradecylphosphonium bromide	Cytec Ind
Cyphos IL101	Trihexyl tetradecylphosphonium chloride	Cytec Ind
TBP	Tri-n-butyl phosphate	Fluka
Primene JMT	Mixture of t-alkyl primary amines	Rohm and Haas
Cyanex 923	Mixture of tri-n-alkyl phosphine oxides	Cytec Ind
Aliquat 336	Tri-octyl methylammonium chloride	Fluka
MIPK	Isopenthyl-methylketone	Fluka
2-Ethyl-1-hexanol	Alcohol	Fluka

Transport experiments were carried out in a two-compartment cell, one compartment each for the (200 cm^3^) source and (200 cm^3^) receiving phases, with a membrane support separating the two aqueous phases. The source and receiving solutions were mechanically stirred by means of four blade impellers (11.5 cm diameter). Indium(III) permeability (P) was estimated by the use of the common relationship:

12 $$\ln \frac{{\left[ {In} \right]_{s,t} }}{{\left[ {In} \right]_{s,0} }} = - \frac{A}{V}Pt$$

where [In]_s,t_ and [In]_s,0_ are the indium concentrations in the source phase at a time during the experiment and at time zero, respectively; A is the effective membrane area (11.3 cm^2^); V is the volume of the source phase; and t is the elapsed time. The percentage of indium recovered in the receiving phase was calculated as:

13 $$\% = \frac{{\left[ {In} \right]_{s,0} - \left[ {In} \right]_{s,t} }}{{\left[ {In} \right]_{r,t} }}x100$$

where [In]_r,t_ is the indium concentration in the receiving phase at a certain time, and [In]_s,0_ and [In]_s,t_ have the same meaning as above. Indium was analysed in the source and receiving phases by atomic absorption spectrometry. The In(III) concentration in the aqueous phases was found to be reproducible to ± 3%.

The membrane support used in the investigation was a Millipore Durapore GVHP4700, with 75% porosity (ε, defined as the ratio between the volume of the pores and the total volume of the membrane), 12.5 × 10^–3^ cm thickness (d_org_), 1.67 tortuosity (τ, a property of the porous membrane support in relation to the turns in the pore channels) and 2.2 × 10^–5^ cm pore size. The Millipore Durapore HVHP4700 support had the same specifications as above but with a 4.5 × 10^–5^ cm pore size. Both were composed of polyvinylidene difluoride. The liquid membranes were prepared by immersion of the support in a carrier solution for 24 h and letting it drip for 20 s before being placed in the cell.

## Conclusions

This investigation demonstrated that the ionic liquid HA324H^+^Cl^-^ is efficient for the transport of indium(III) from hydrochloric acid solutions. It was experimentally found that the best conditions for indium(III) transport were 750 and 500 min^-1^ stirring speeds in the source and receiving phases, respectively; 1–2 M HCl in the source phase and a carrier concentration of 0.23 M. In terms of metal transport, this carrier compares well with other carriers of various types, though indium(III) recovery in the receiving solution was not the best compared with that for other carriers, i.e., phosphonium salts and the phosphine oxide Cyanex 923. The kinetic model for indium(III) permeation showed that the permeation process is controlled by diffusion of the InCl4- species across the source phase layer and diffusion of the In(III)-carrier complex across the liquid membrane, with the former dominant when the carrier concentration in the membrane phase is low. The results derived from this work could be improved upon by the use of a smart membrane device, i.e., hollow fibre modules, and smart liquid membrane operation, i.e., pseudoemulsion-based hollow fibre strip dispersion technique.
